# Characterization of photoreceptor degeneration in the rhodopsin P23H transgenic rat line 2 using optical coherence tomography

**DOI:** 10.1371/journal.pone.0193778

**Published:** 2018-03-09

**Authors:** Natsuki Monai, Kodai Yamauchi, Reiko Tanabu, Takayuki Gonome, Sei-ichi Ishiguro, Mitsuru Nakazawa

**Affiliations:** 1 Department of Ophthalmology, Hirosaki University Graduate School of Medicine, Hirosaki, Japan; 2 Department of Ophthalmology, Tohoku University Graduate School of Medicine, Sendai, Japan; University of Florida, UNITED STATES

## Abstract

**Purpose:**

To characterize the optical coherence tomography (OCT) appearances of photoreceptor degeneration in the rhodopsin P23H transgenic rat (line 2) in relation to the histological, ultrastructural, and electroretinography (ERG) findings.

**Materials and methods:**

Homozygous rhodopsin P23H transgenic albino rats (line 2, very-slow degeneration model) were employed. Using OCT (Micron IV^®^; Phoenix Research Labs, Pleasanton, CA, USA), the natural course of photoreceptor degeneration was recorded from postnatal day (P) 15 to P 287. The OCT images were qualitatively observed by comparing them to histological and ultrastructural findings at P 62 and P 169. In addition, each retinal layer was quantitatively analyzed longitudinally during degeneration, compared it to that observed in wild type Sprague-Dawley (SD) rats. The relationships between the ERG (full-field combined rod-cone response, 3.0 cds/m^2^ stimulation) findings and OCT images were also analyzed.

**Results:**

In the qualitative study, the two layers presumably corresponding to the photoreceptor inner segment ellipsoid zone (EZ) and interdigitation zone (IZ) were identified in the P23H rat until PN day 32. However, the photoreceptor inner and outer segment (IS/OS) layer became diffusely hyperreflective on OCT after P 46, and the EZ and IZ zones could no longer be identified on OCT. In contrast, in the SD rats, the EZ and IZ were clearly distinguished until at least P 247. The ultrastructural study showed partial disarrangements of the photoreceptor outer segment discs in the P23H rats at P 62, although a light-microscopic histological study detected almost no abnormality in the outer segment. In the quantitative study, the outer retinal layer including the outer plexiform layer (OPL) and the outer nuclear layer (ONL) became significantly thinner in the P23H rats than in the SD rats after P 71. The thickness of the IS/OS layer was maintained in the P23H rats until P 130, and it became statistically thinner than in the SD rats at P 237. The longitudinal attenuation in the amplitude of the a- and b-waves of ERG was significantly correlated with the thickness of the combined OPL and ONL but not with that of the IS/OS layer.

**Conclusion:**

OCT showed the degenerated photoreceptor IS/OS layer in rhodopsin P23H transgenic rats (line 2) as a diffuse hyperreflective zone, even in the early stage, with the partially disarranged and destabilized OS discs recognizable by ultrastructural assessment but not by a histological study. The amplitude of the a- and b-waves mainly depends on the thickness of the OPL and ONL layer rather than the thickness of the photoreceptor IS/OS layer in P23H rats.

## Introduction

Retinitis pigmentosa (RP) is a clinical entity caused by mutations in more than 60 genes that have been previously identified and reported (RetNetTM, Retinal Information Network: http://sph.uth.edu/retnet/home.htm). RP is the most frequently encountered hereditary retinal photoreceptor degenerative diseases, and the overall prevalence has been reported to be 1 in 4,000–5,000 people worldwide [1]. Mutations in the rhodopsin gene are found as the most common cause of the autosomal dominant type of RP (adRP) [1, 2]. The point mutation P23H was first identified as a causative mutation for adRP^3^ and is the most frequently identified mutation among patients with adRP in the US [3]. The clinical appearance associated with the P23H mutation has been characterized as a mild form of RP, although there is some phenotypic variability [4, 5].

Recent advances in optical coherence tomography (OCT) technology have clarified a number of previously unknown morphological details regarding various retinal diseases, including RP [6–19]. The advantage of OCT includes its non-invasiveness and repeatability, and OCT is not plagued by the artifacts sometimes seen during processing in histological sections [20]. Evaluating RP patients’ retinal morphology by analyzing OCT images, such as monitoring the level of photoreceptor damage and/or the effectiveness of treatment may therefore prove extremely useful in the future.

One of problems in the clinical treatment of RP is the genetic heterogeneity of the disease, which induces different types of photoreceptor cell death and subsequently results in phenotypic variability. Therefore, to understand the detailed mechanisms underlying photoreceptor degeneration and to develop an effective treatment for RP, a mutation-specific analysis is needed. The OCT findings of RP may also be heterogeneous, as the retinal morphology in RP is affected by its heterogeneous mechanisms of photoreceptor degeneration associated with its varied genetic background. Characterizing the details of OCT findings for RP will therefore likely require consideration of the genetic heterogeneity of RP.

The OCT findings of retinal degeneration in heterozygous rhodopsin P23H transgenic rats (line 1), retinal degeneration (rd) 10 and rd 12 mice, arrestin knock-out mice, and Royal College of Surgeons (RCS) rats have been reported [21–26]. Rhodopsin P23H transgenic rats were generated using a mouse rhodopsin P23H transgene with wild-type (wt) Sprague-Dawley (SD) rat. [27] Three lines are known; line 1 (fast degeneration model), line 2 (very-slow degeneration model), and line 3 (slow degeneration model) [27]. Although the OCT findings in P23H rats (line 1) have been reported, those in P23H rats (line 2) have yet to be described. Because patients harboring the rhodopsin P23H mutation generally present with mild and slowly progressive type of RP, we believe it valuable to provide information regarding the OCT findings in the P23H rats (line 2) in order to apply these results to the clinical fields.

We therefore qualitatively and quantitatively characterized OCT images in homozygous rhodopsin P23H transgenic albino rats (line 2) with regard to histological, ultrastructural, and electroretinography (ERG) assessments.

## Materials and methods

### Experimental animals

Experimental procedures in the present study conformed to the Association for Research in Vision and Ophthalmology (ARVO) Statement for the Use of Animals in Ophthalmic and Vision Research and were approved by the Committee for Ethics in animal experiments of Hirosaki University Graduate School of Medicine (Approval Number: M11026). The homozygous rhodopsin P23H transgenic rats (line 2) were generously provided by Dr. Mathew M LaVail of the University of California. The SD rats were purchased from Clea Japan (Tokyo, Japan) and used as wt control rats. The animals were maintained at the Hirosaki University Graduate School of Medicine Animal Care Service facility under a cycle of 12 h of light (50 lx illumination) and 12 h darkness (<10 lx environmental illumination). Care was taken not to cause light-induced photoreceptor damage. Food and water were available *ad libitum*.

### OCT examinations

OCT was performed via the previously reported method with slight modifications [25]. In brief, using a Micron^®^ IV (Phoenix Research Labs, Pleasanton, CA, USA) with a contact lens specifically designed for mouse OCT, OCT was carried out at 17 time points starting from the postnatal day (P) 15 until P294 for P23H rats and at 6 points from P26 until P247 for SD rats. Although we employed a contact lens designed for rats in previous experiments, we found that the contact lens designed for mice provided a better resolution and clearer images, even for rats, in the OCT [25]. We therefore employed the contact lens designed for mice in the present study. Two to four rats (four to eight eyes) were evaluated at each time point. In addition, to keep the rats’ cornea sufficiently clear during the OCT examination, we prepared four different age groups of the P23H rats and two age groups of SD rats (three to six rats in each group) and performed OCT measurements alternately using these groups of rats (two to three times per group). Rats were anesthetized with an intraperitoneal injection of a mixture of medetomidine hydrochloride (0.315mg/kg), midazolam (2.0mg/kg), and butorphanol tartrate (2.5mg/kg). To prevent any pain associated with injections, rats were first anesthetized by inhalation of 80% carbon dioxide and 20% oxygen prior to the intraperitoneal injection. The physical conditions, including heartbeat and respiratory pattern, of rats were frequently monitored during experiments by inspection and gentle palpation by the researchers. Pupils were dilated by instillation of a mixture of 0.5% tropicamide and 0.5% phenylephrine hydrochloride eyedrops. Corneal surface was protected using a 1.5% hydroxyethylcellulose solution. The rat ocular fundus was simultaneously monitored using a fundus camera, and the position of the retinal OCT image was set horizontally at 1 disc diameter superior to the optic disc. Fifty images were averaged to eliminate the projection artifacts. The acquired OCT images were quantitatively analyzed using the InSight^®^ software program (Phoenix Research Labs). Three to eight images from two to five rats each from the both P23H rat and SD rat groups were selected based on the quality of the pictures in terms of the image sharpness at each time point in order to perform as precise a segmentation analysis using InSight^®^ as possible. The pictures deemed unsuitable for the segmentation analysis because of blurring from rats’ respiratory body movement during OCT experiments were eliminated.

### Retinal layer thickness analysis (segmentation)

**Fig 1:**
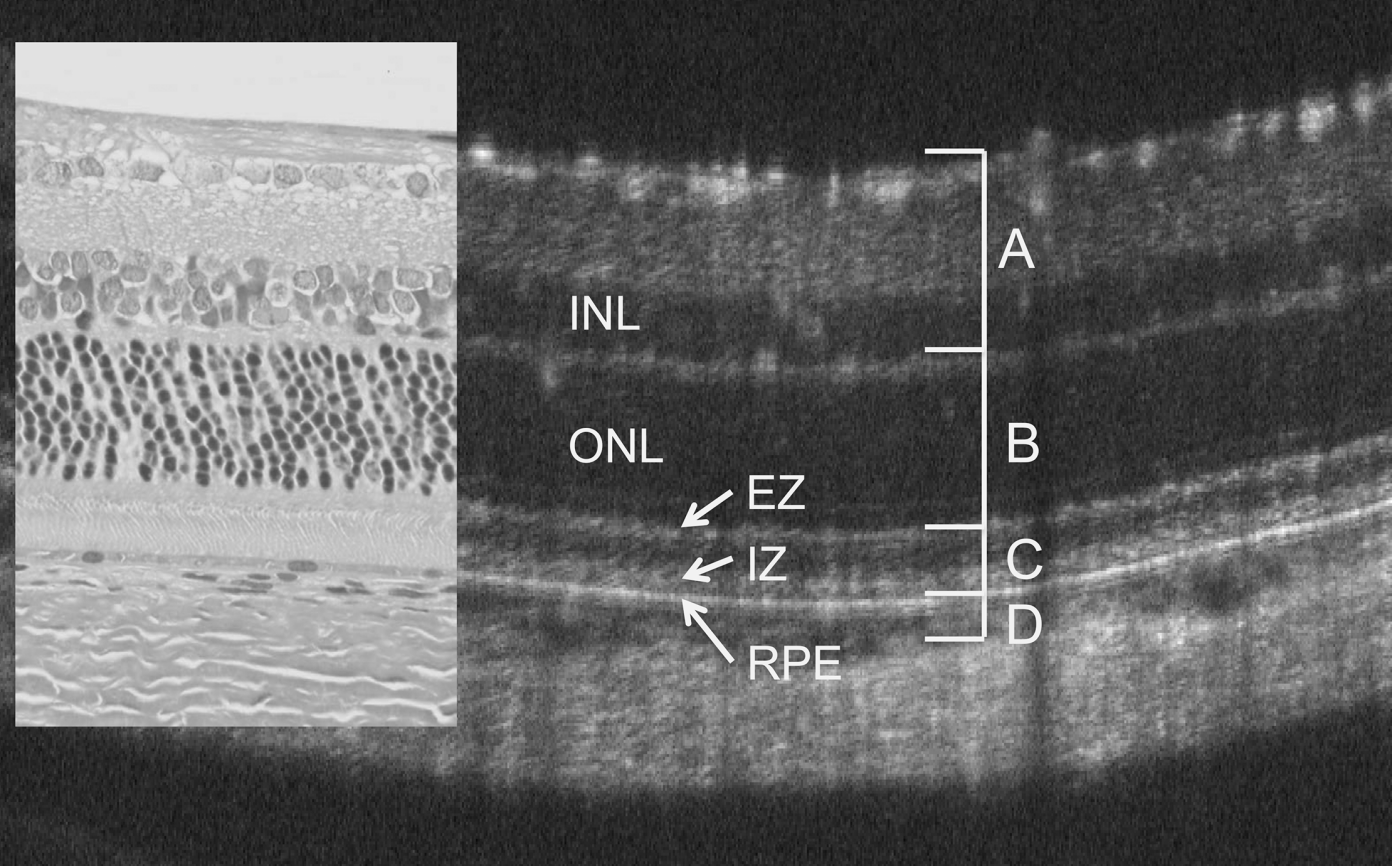
Relationship between the image on optical coherence tomography (OCT, P33) and a histological section (hematoxylin and eosin, HE, P 69) of Sprague-Dawley (SD) rat retina. The retinal layers, including the inner nuclear layer (INL), the outer nuclear layer (ONL), the inner segment (IS) ellipsoid zone (EZ), the interdigitation zone (IZ), and the retinal pigment epithelium (RPE), can be identified on OCT. The retinal sublayers are defined as A, B, C, and D.

We measured the thicknesses of the inner (Fig 1A), middle (Fig 1B), and outer (Fig 1C) layers of the neural retina and the combined retinal pigment epithelium (RPE) and choroid layers (Fig 1D). The inner layer (A) includes the retinal nerve fiber layer (NFL), ganglion cell layer (GCL), inner plexiform layer (IPL), and inner nuclear layer (INL); the middle layer (B) consists of the combined outer plexiform and outer nuclear layers (OPL and ONL, respectively); and the outer layer (C) consists of the photoreceptor inner segment (IS) and outer segment (OS) layer (Fig 1).

Segmentation was performed by the method previously described using the InSight^®^ software program with slight modifications [25]. In brief, the borderlines between each retinal sublayer (A-D) were first defined using the OCT pictures. These borderlines were initially automatically segmented by the InSight^®^ software program, and then manually corrected by the researchers if necessary. The average distance (μm) between each borderline was then calculated using the raw data (evenly distributed about 1,000 points throughout each OCT image) from the InSight^®^ software program. Because Ryals et al. reported that there was no difference in the progression of retinal degeneration between left and right eyes of the same rat [26], we employed the mean value of both eyes and counted it as one observation. The data obtained from the P23H rats at P71 and P72 and at P237 and P287 were combined for statistical calculations. The overall average retinal layer thickness was presented as the mean ± standard error and the mean ± standard deviation (S1 Dataset).

### ERG examination

Because the rat retina consists of both rod and cone photoreceptors with no macular structure, we deemed scotopic full-field combined rod-cone ERG suitable for the comparison of ERG and OCT images. Combined rod-cone ERG was performed as previously described with slight modifications [25]. In a previous study using RCS rats, we confirmed that the amplitudes of both the a- and b-waves increased in a stimulus-dependent manner by changing the stimulation from 3.0 to 30.0 cd.s/m^2^ [25], in accordance with the International Society for Clinical Electrophysiology of Vision standards for the combined rod-cone ERG [28]. We therefore fixed the light stimulus at 3.0 cd.s/m^2^ in the present study. ERG was carried out at 6 time points starting from P21 until P294 for P23H rats and at 6 time points from P22 to P247 for SD rats. Rats were dark-adapted for 24 h and then anesthetized according to the same method described above. The physical conditions were also monitored as described above. A reference electrode was subcutaneously placed in the center of the scalp, and a ground electrode was placed in the proximal portion of the tail skin. During the measurement, the body temperature was maintained at 37°C, using a body warmer. Pupils were dilated with a mixture of 0.5% tropicamide and 0.5% phenylephrine hydrochloride eye drops. After the corneal surface was anesthetized using 0.4% oxybuprocaine hydrochloride eyedrops, a contact-lens electrode (Micron^®^ Ganzfeld ERG; Phoenix Research Labs) was applied directly to the corneal surface. Twenty responses (stimulus interval = 10 s) were acquired and averaged to record the standard waveform. The amplitude of the a-wave was defined as the difference between the base line potential and the peak of the a-wave. The amplitude of the b-wave was defined as the difference between the peak of the a-wave and the peak of the highest positive wave after the a-wave. Similarly to the OCT measurement, we employed the average value of the amplitudes obtained from both eyes of the same rats and counted it as one observation. The data obtained from P21 and P22 of the P23H rats were combined. The amplitudes of both the a- and b-waves (S2 and S3 Datasets) were statistically analyzed.

### Histological examination

Histological examinations were performed using eyes excised from P23H rats at P62 and P169 and SD rat at P69. Immediately after euthanasia by 100% carbon dioxide inhalation, the eyeballs were enucleated under a microscope, and an aliquot of 2% glutaraldehyde and 2% paraformaldehyde solution (pH 7.4) was injected into the vitreous space through the ciliary body portion to avoid retinal detachment potentially occurring during further tissue processing. The eyeballs were fixed in 2% glutaraldehyde and 2% paraformaldehyde solution (pH 7.0) for 2 h at room temperature. Subsequently, the eyeballs were re-fixed in 4% paraformaldehyde solution (pH 7.0) for 24h at 4°C. The eyes were embedded in paraffin and cut into 6-μm thick sections, horizontally at the level of 1 disc diameter superior to the optic disc, which was intended to be the position identical to the OCT measurement. The excised tissue was stained with hematoxylin and eosin (HE) and was photographed under a light microscope (DP-71; Olympus, Tokyo, Japan). Histological findings were compared to the corresponding findings from OCT images.

### Electron microscopic examinations

Electron microscopic examinations were performed using eyes excised from P23H rats at P62 and P169 and SD rat at P 9. Immediately after enucleation as described above, the eyes were fixed with 2.5% glutaraldehyde and 2% paraformaldehyde solution (pH 7.4) for 24 h at 4°C. Similarly to histological processing, to avoid retinal detachment potentially occurring during tissue processing, an aliquot of 2.5% glutaraldehyde and 2% paraformaldehyde solution (pH 7.4) was injected into the vitreous space through the ciliary body portion. The retina and choroid were dissected out, post-fixed in phosphate buffered 1% osmium tetroxide (pH 7.4) for 3 h at 4°C dehydrated in an ascending series of ethanol solutions (50%-100%). The blocks were embedded in epoxy resin. Thin sections (t = 80–90 nm) were stained in uranyl and lead salt solutions. The sections were photographed using transmission electron microscopy (H-7600, Hitachi, Tokyo, Japan) at 100kV.

### Statistic examination

All of the statistical analyses were performed using the SPSS software program version 22 (Statistical Package for the Social Sciences, Chicago, IL, USA). The data from the two groups were compared using a two-way repeated analysis of variance (two-way repeated ANOVA) after the normality of each distribution was confirmed by the Shapiro-Wilk test. *Post hoc* analyses were performed using Tukey’s *t*-test between similar age-groups (P23H vs. SD: P26 vs. P26, P32 vs. P33, P46 vs. P54, P71-72 vs. P82, P89 vs. P82, P125 vs. P134, and P237-287 vs. P247, respectively) for OCT segmentation. Student’s *t*-test was used to compare the amplitude of ERG between the two groups (two pairs of age-matched rat groups; P23H vs. SD: P22 vs. P22, P99 vs. P92, and P253 vs. P247, respectively). In addition, the correlation between the thickness of each retinal layer and the amplitude of the ERG a- and b-waves was examined using a Pearson’s correlation analysis followed by a step-wise multivariate regression analysis. *P* values < 0.05 were considered statistically significant.

## Results

### OCT findings in SD rats

**Fig 2:**
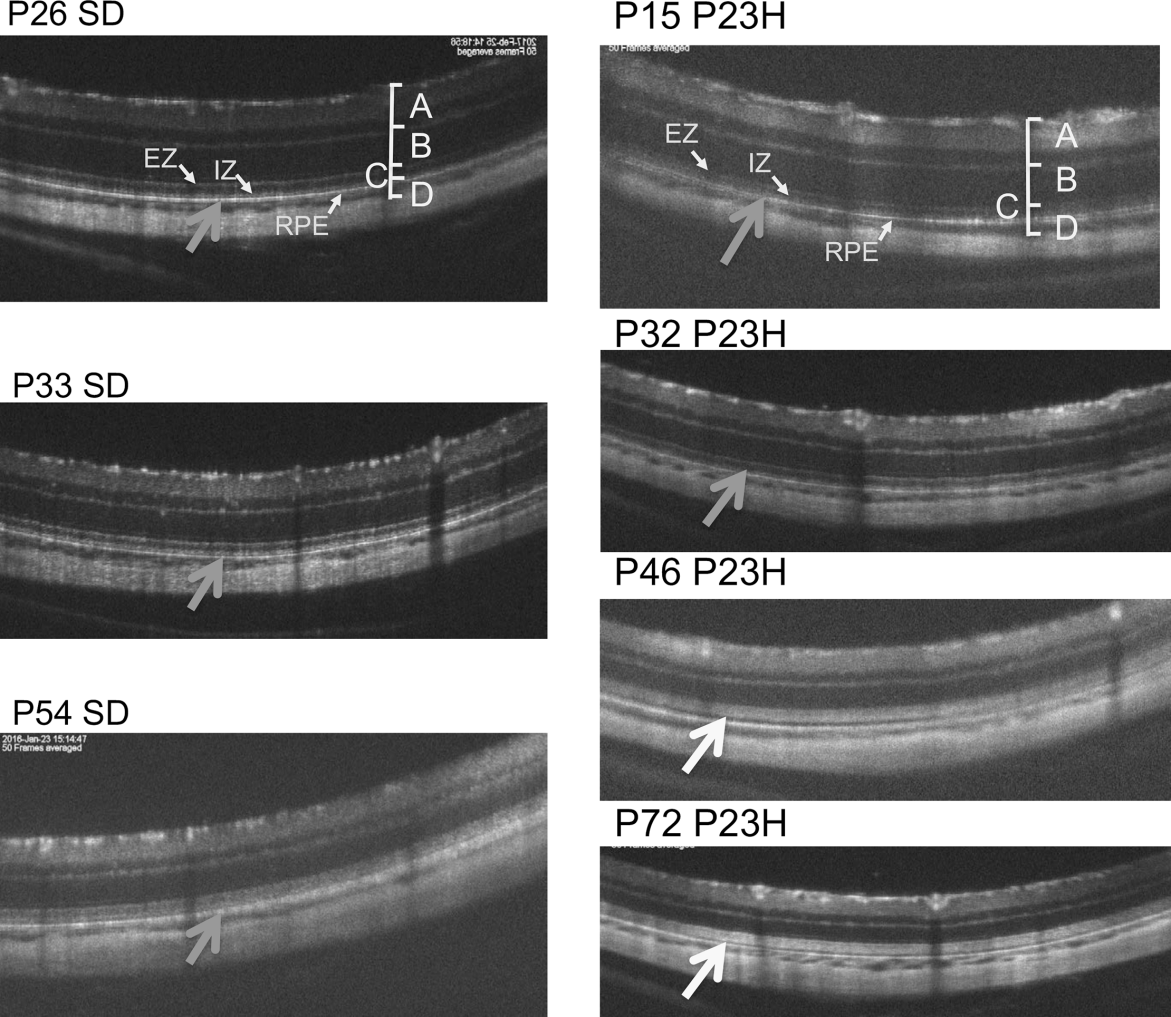
The natural course and longitudinal changes on OCT images of SD rat (from P26 to P54, left side) and P23H transgenic rat (line 2) (from P15 to P72, right side) retinas. In P23H rats, the IS/OS layer becomes diffusely hyperreflective after P46. The retinal sublayers defined as A, B, C, D, EZ, IZ, and RPE are identical to those in Fig 1.

**Fig 3:**
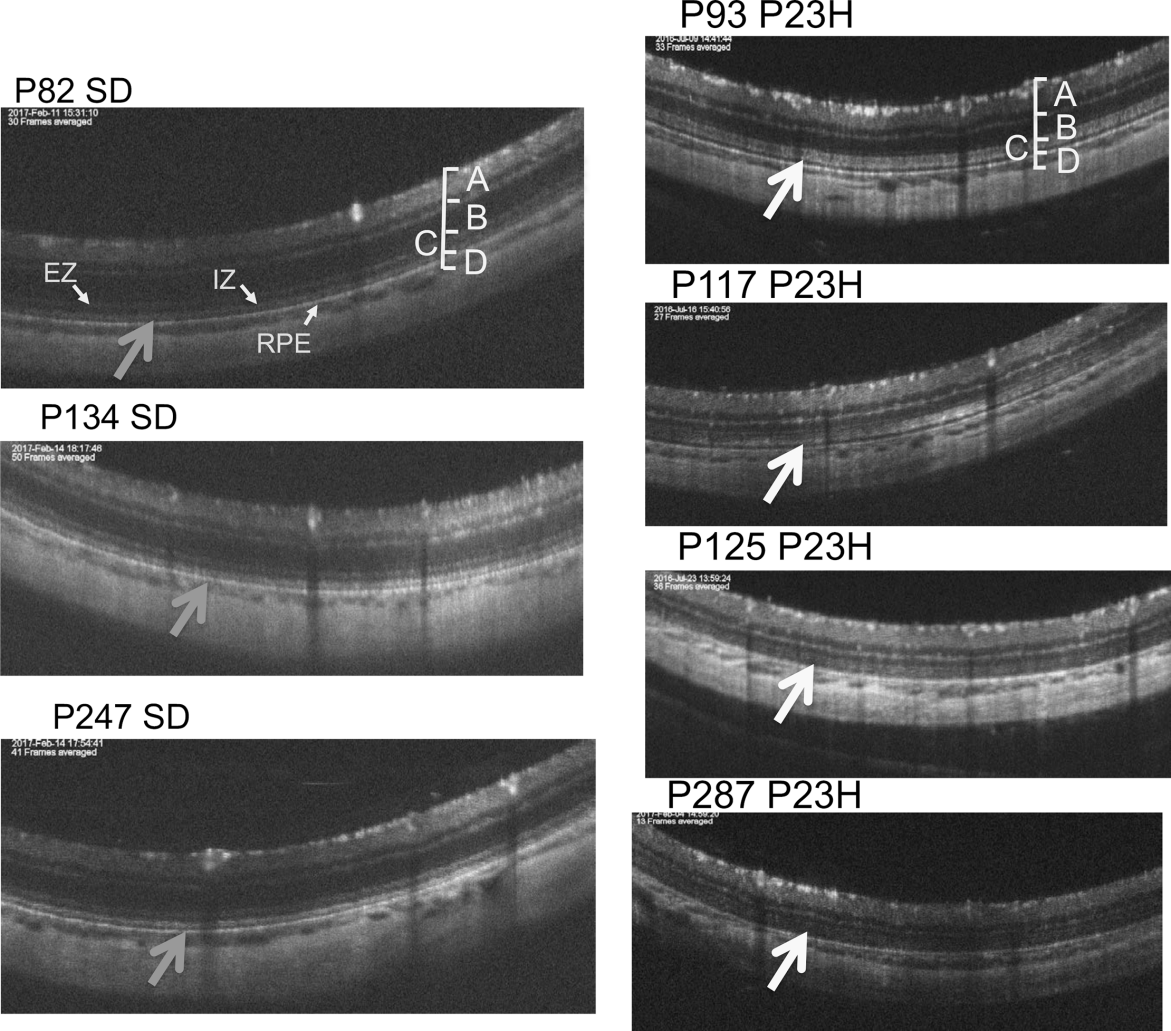
The natural course and longitudinal changes on OCT images of SD rat (from P82 to P247, left side) and P23H transgenic rat (line 2) (from P93 to P287, right side) retinas. In P23H rats, the IS/OS layer became diffusely hyperreflective and layer B became attenuated. The retinal sublayers defined as A, B, C, D, EZ, IZ, and RPE are identical to those in Fig 1.

Fig 1 shows a typical OCT image of a P33 SD rat, as well as the corresponding image of an HE-stained histological section of an SD rat at P69. Similarly to the wt RCS^+/+^ rats previously described [25], the retinal structures were clearly identified on OCT images: the NFL, GCL, IPL, INL, OPL, ONL, photoreceptor IS and OS (IS/OS) layers, RPE layer, choroid, and sclera. In addition, two distinct hyperreflective bands, corresponding to the IS ellipsoid zone (EZ) and the interdigitation zone (IZ), were consistently visualized on OCT in the SD rats. The EZ and IZ have been defined in the human and mouse OCT images [21, 24, 29]. In Figs 2 and 3, these basic retinal structures consistently appeared on OCT of P26 to P247 in the SD rats.

### Qualitative analyses of the OCT findings from the rhodopsin P23H transgenic rats (line 2)

**Fig 4:**
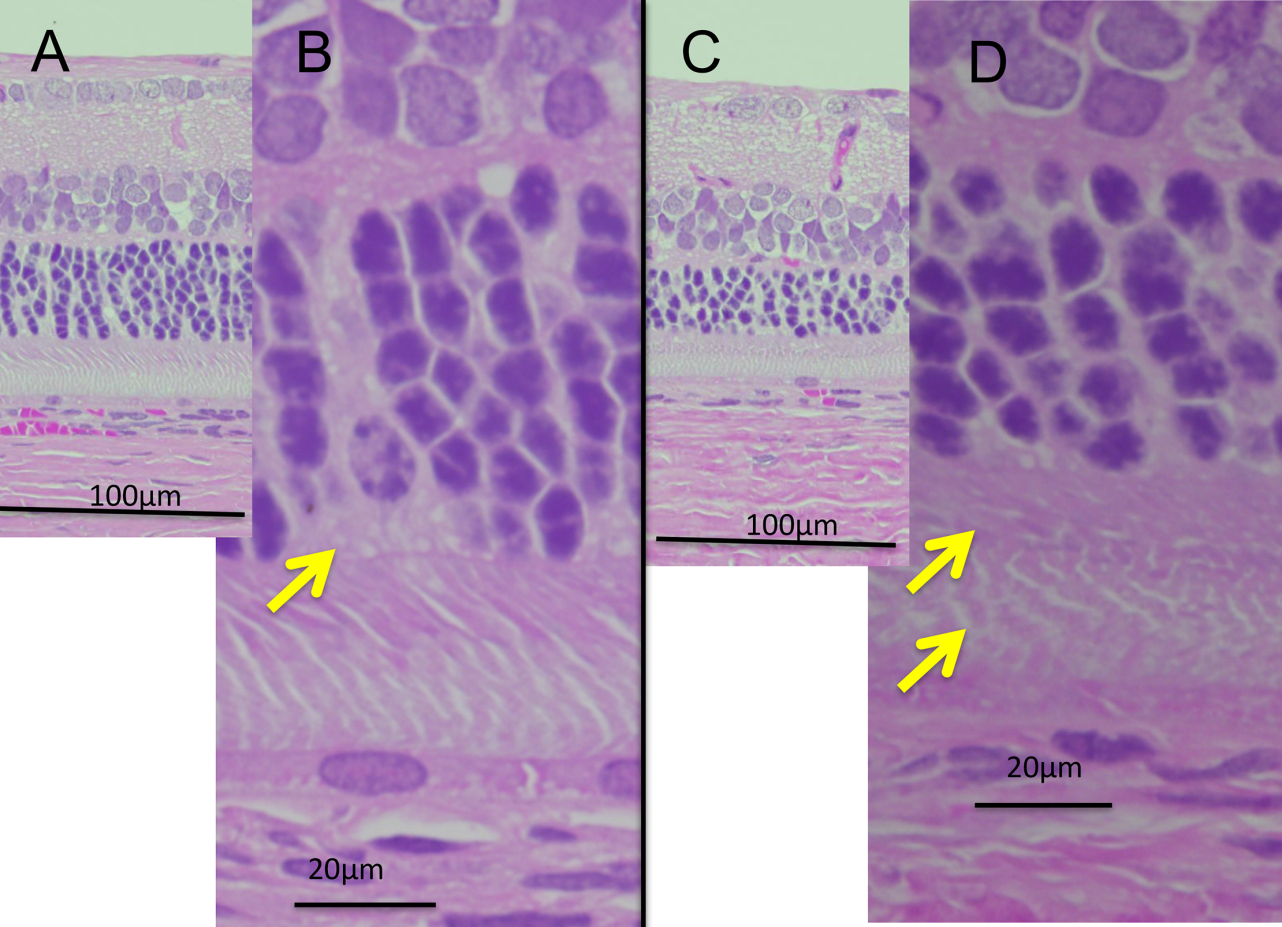
Histologic findings of the P23H rat (line 2) retinas. A and B (high magnification), HE sections of P23H rat retina at P62. Although the OS discs appear intact, vacuolations are found in the ONL, indicating the early phase of photoreceptor degeneration (arrow). C and D (high magnification), HE sections of P23H rat retina at P169. Obvious disarragments can be seen in the IS/OS layer (arrows).

**Fig 5:**
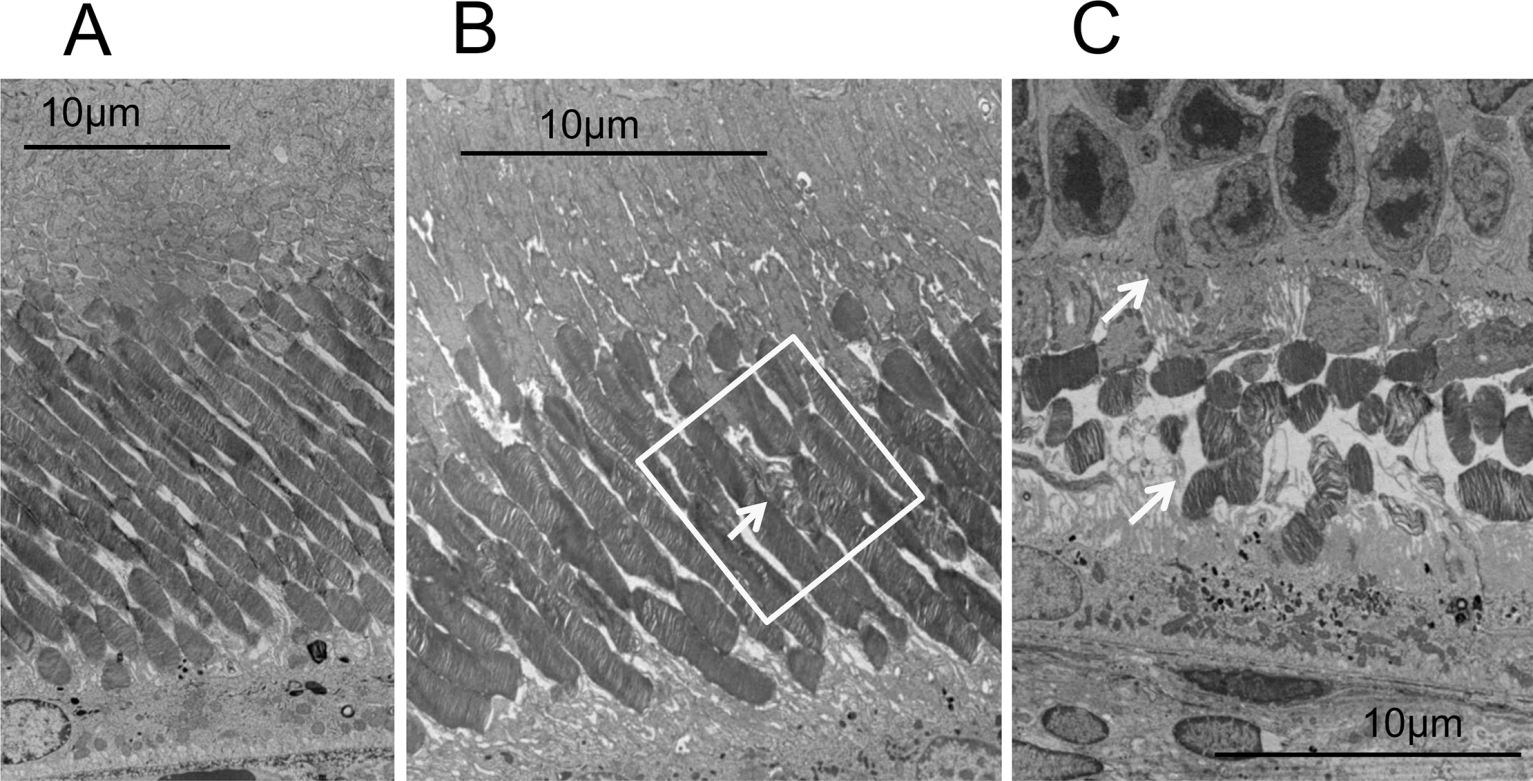
Electon microscopy findings for the SD and P23H (line 2) rat retinas. A, no apparent abnormalities were found in the IS/OS layer of the SD rat retina at P69. B, disorganized OS discs were found (yellow rectangle and an arrow) in the P23H rat retina at P62. C, the IS/OS layer was severely degenerated (arrows) in the P23H rat retina at P169.

Similarly, we analyzed the OCT images of the P23H rats (line 2) to characterize the longitudinal changes that occur during retinal degeneration. Figs 2 and 3 show the characteristic OCT findings of P23H rats. In the early stage of degeneration, at P15 and P32, the basic retinal structure appears to be preserved, similarly to the wt SD rats with the EZ and IZ layers clearly identified on OCT (Fig 2). However, instead of these distinctive two bands, the photoreceptor IS/OS layer became diffusely hyperreflective after P43, and these findings were similarly conserved until P294. Furthermore, on comparing these findings were compared to the histological and ultrastructural findings obtained at P62 and P169 (Figs 4 and 5), although vacuolation, indicating early-stage photoreceptor degeneration was seen in the ONL at P42, no apparent abnormalities were found in the photoreceptor IS/OS layer on an HE section (Fig 4A and 4B). Although no apparent abnormalities were found in the IS/OS layer at P69 in the SD rat on electron microscopy (Fig 5A), disarrangements of the OS discs were detected in the same layer even at P62 in the P23H rat (Fig 5B). At P162, disarrangements of the IS and OS layers and a decrease in the ONL thickness were detected on an HE section (Fig 4C and 4D). Severe degeneration in the IS and OS layers was obvious on electron microscopy (Fig 5C). By comparing these findings to the OCT images, we concluded that the diffuse hyperreflective zone appearing in the IS/OS layer after P43 corresponded to early changes in the disarrangement of the OS discs, which cannot be detected by HE staining and can only be observed on electron microscopy.

### Quantitative (retinal thickness) analyses of OCT findings

**Fig 6:**
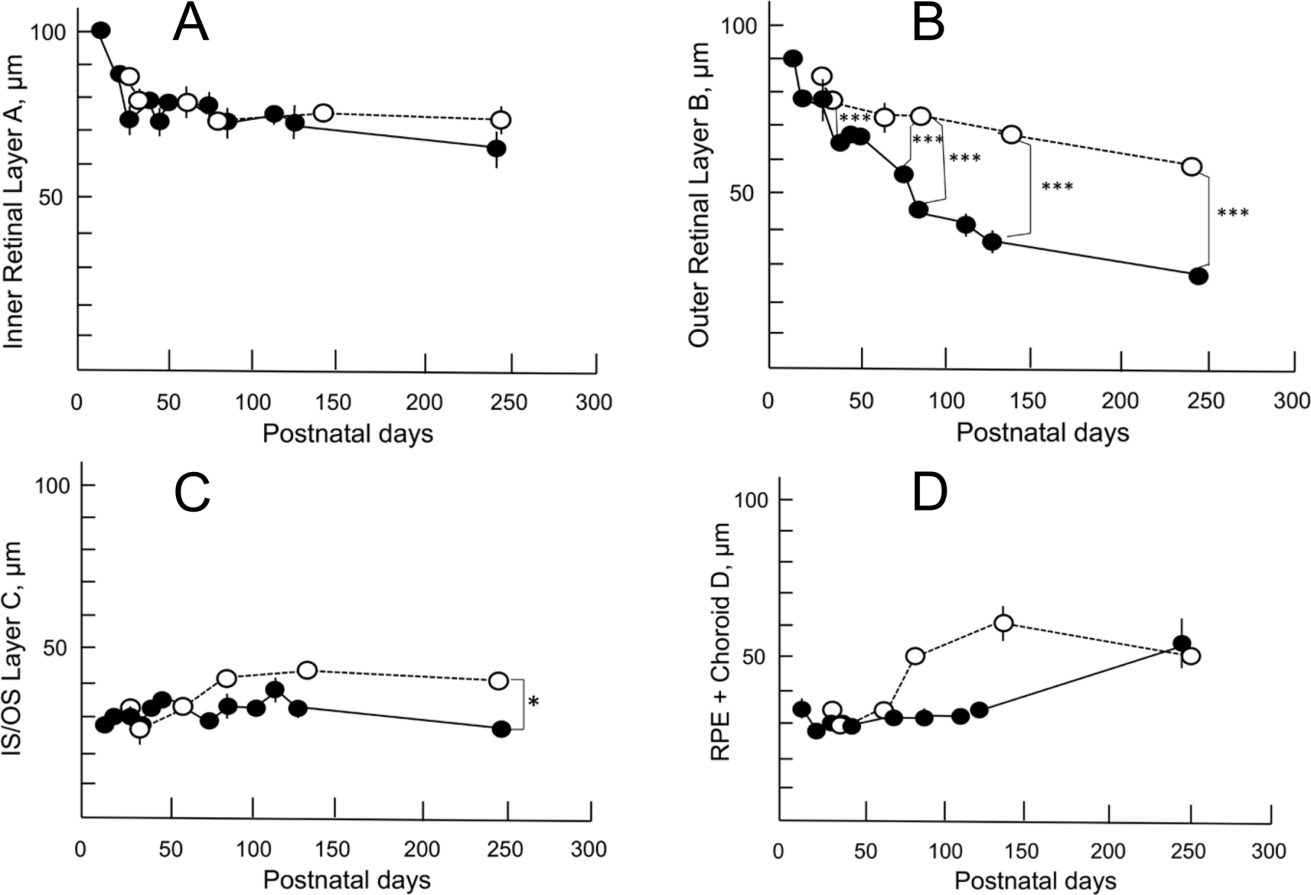
Longitudinal changes in the thickness of the retinal sublayers on OCT in P23H (closed circle) and SD (open circle) rats. A, changes in the thickness of the retinal sublayer A, which consists of the retinal nerve fiber layer, retinal ganglion cell layer, the inner plexiform layer, and the INL. B, changes in thickness of retinal sublayer B, which consists of the OPL and the ONL. C, changes in thickness of retinal sublayer C, which consists of the IS/OS layer. D, changes in thickness of the retinal sublayer D, which consists of the RPE and choroid. Tukey’s *t*-test was performed for statistical comparisons between the two groups. Significance level: **, *P* < 0.01; ***, *P* < 0.001). Values are indicated by mean ± standard error.

Fig 6 shows the time course of the change in thickness of each retinal layer in both rhodopsin P23H transgenic rats and SD rats (S1 Dataset). The thickness of the inner retinal layer (retinal layer A) of the P23H rats did not significantly differ from that of SD rats (Fig 6A). In contrast, the combined OPL and ONL (retinal layer B) in the P23H rats underwent rapid thinning after P 71 (Fig 6B, *P* < 0.01). The thickness of the photoreceptor IS/OS layer (retinal layer C) of the P23H rats gradually became thinner and was significantly thinner than that of SD rats only after P 237 (Fig 6C, *P* = 0.022). The average thickness of the combined RPE and choroid (retinal layer D) did not differ significantly between the two groups (Fig 6D).

### Correspondence of OCT images and ERG findings

**Fig 7:**
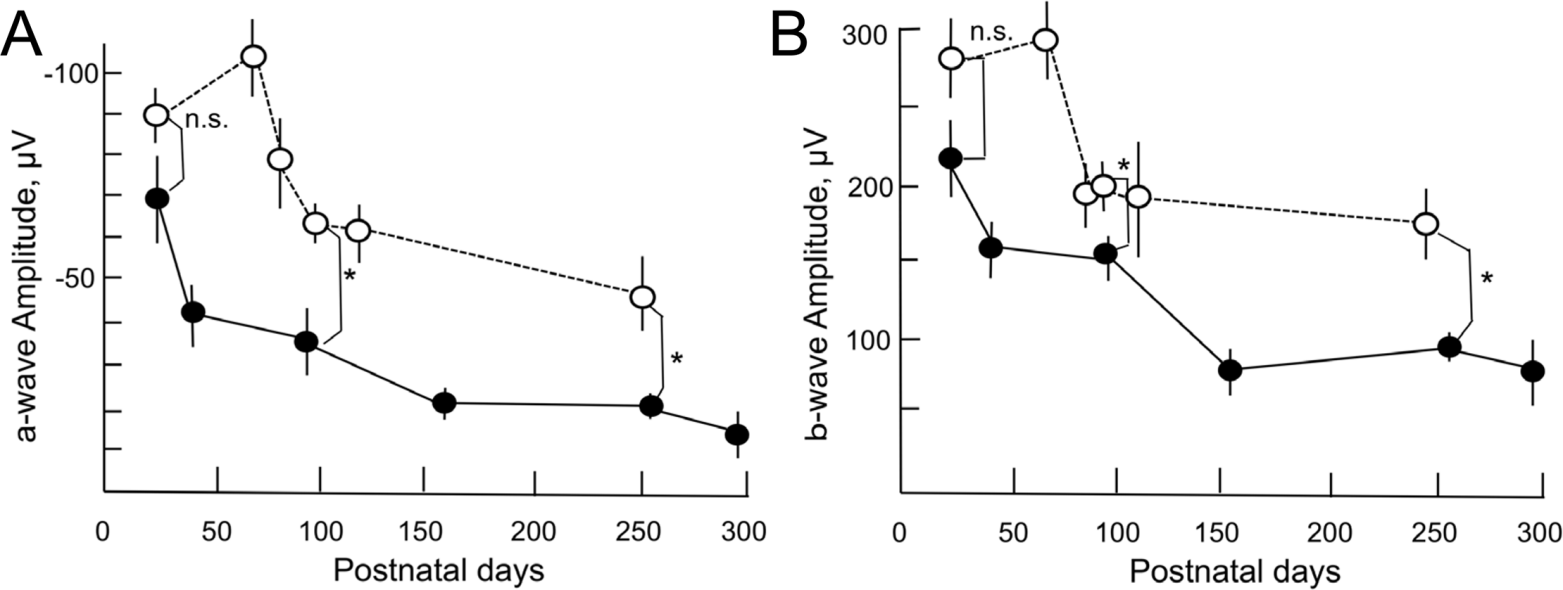
Longitudinal changes in the amplitude of a- (panel A) and b- (panel B) waves of P23H (closed circle) and SD (open circle) rats. Student’s *t*-test was performed for statistical comparisons between the two groups. Significance level: *. P < 0.05. Values are indicated by mean ± standard error.

**Fig 8:**
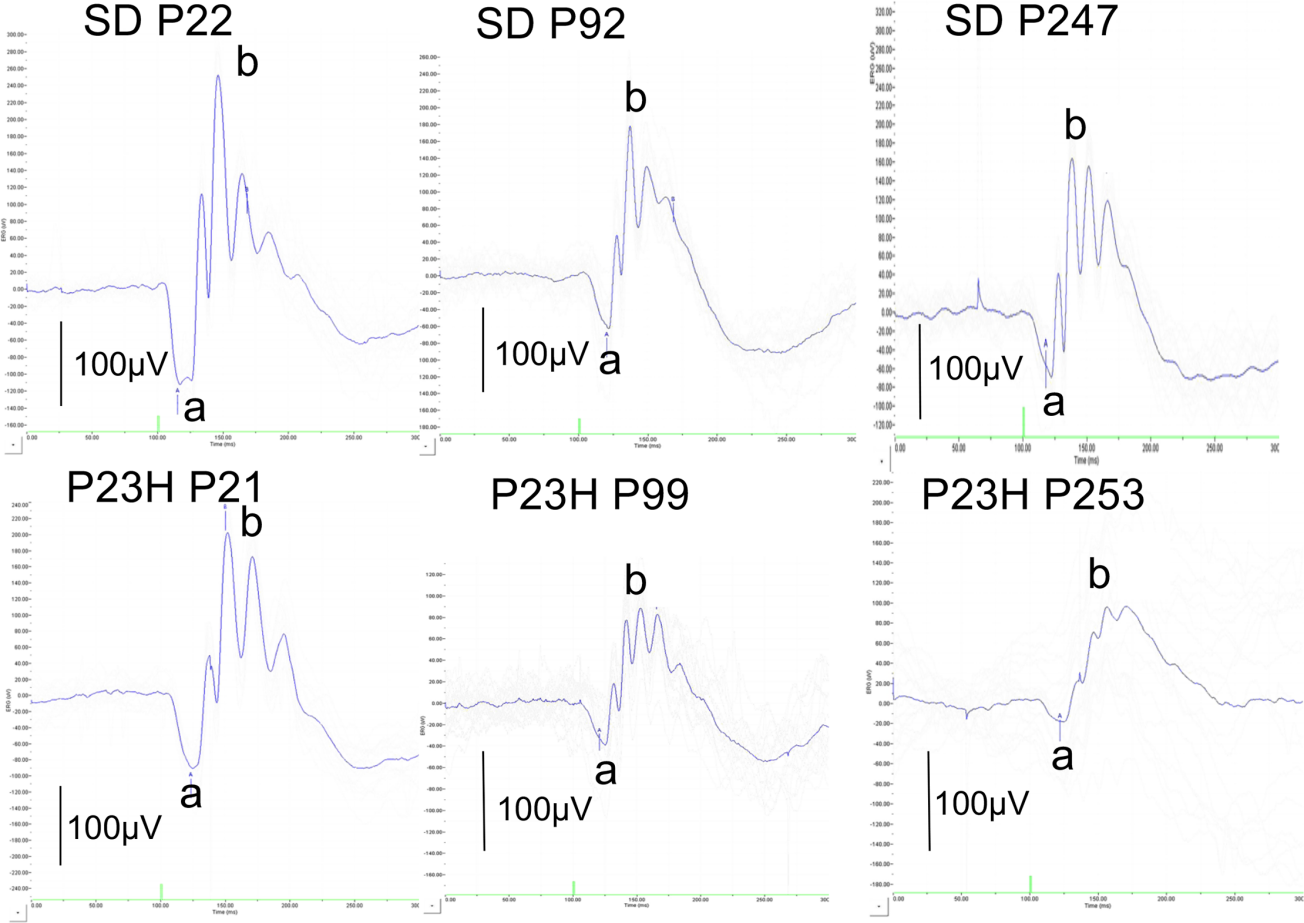
Representative traces of ERG appearances in both SD (P22, P92 and P247) and P23H (P21, P99 and P253) rats.

In SD rats, the amplitude of both a- and b-waves decreased with age (Fig 7); these amplitudes in the P23H rats showed the same tendencies (Fig 7), although the amplitude of the a-waves became significantly lower than in SD rats after P 99 (Fig 7A, S2 and S3 Datasets). Similarly, the P23H rats showed significantly lower amplitude of b-waves than SD rats at P99 and P253 (Fig 7B). The representative traces of both SD (P22, P92, and P247) and P23H (P21, P99, and P253) rats are presented in Fig 8.

**Fig 9:**
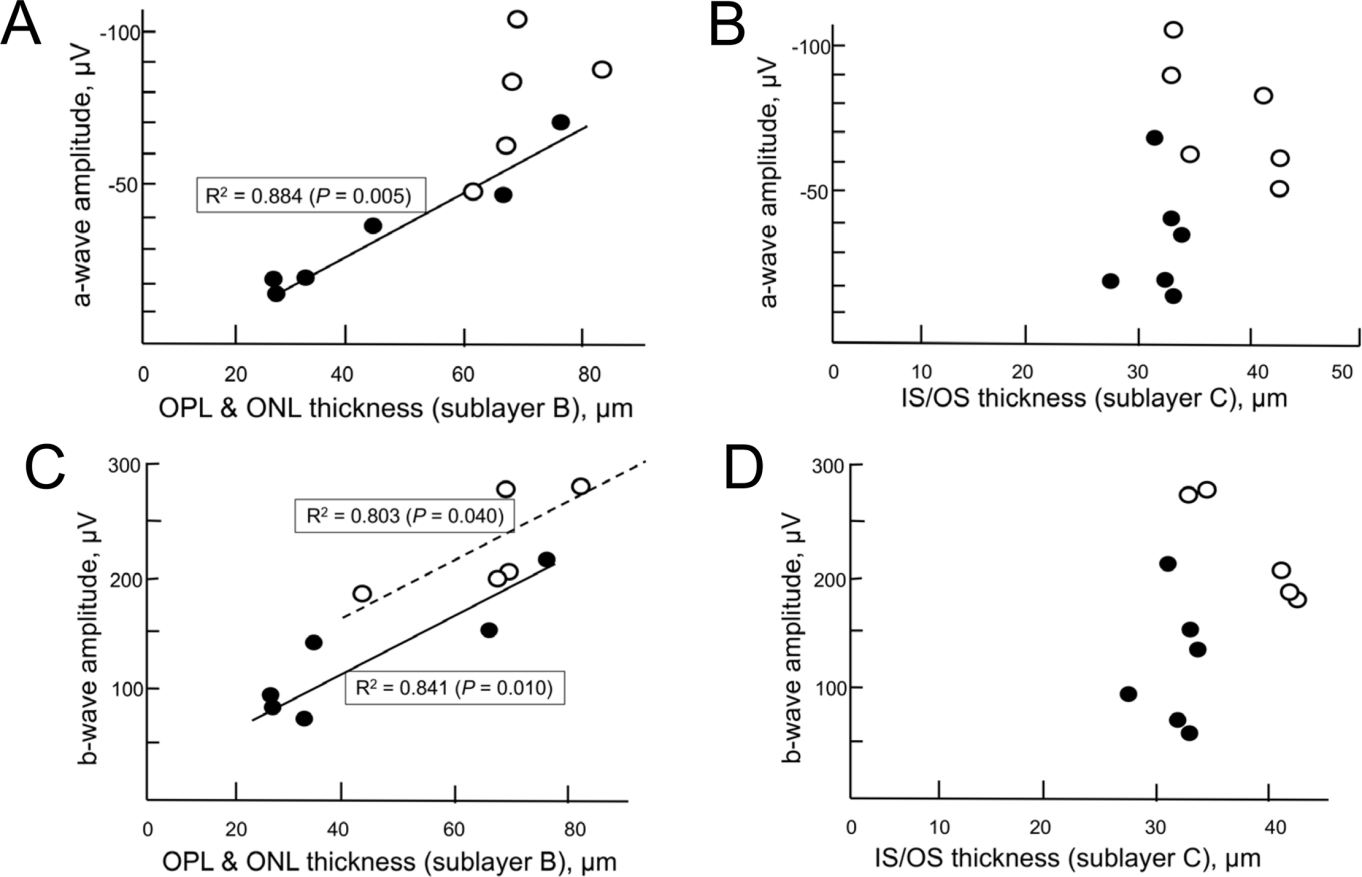
Correlation between the thickness of retinal sublayers B (panel A) and C (panel B) and the amplitude of the a- and b-waves. A, there is a significant correlation between the thickness of sublayer B, which consists of the OPL and the ONL, and the amplitude of the a-wave in P23H rat (closed circle, *R*^2^ = 0.884, *P* = 0.005). B, there is no significant correlation between the thickness of sublayer C, which consists of the IS/OS layer and the amplitude of the a-wave in either P23H or SD rats. C, there are significant correlations between the thickness of sublayer B and the amplitudes of the b-wave in both P23H (closed circle, *R*^*2*^ = 0.841, *P* = 0.010) and SD (open circle, *R*^*2*^ = 0.803, *P* = 0.040) rats. D, there is no significant correlation between the thickness of sublayer C and the amplitude of the b-wave in either P23H or SD rats.

We focused on the quantitative relationships between the thickness of the ONL/OPL (retinal layer B) or IS/OS (retinal layer C) layers and the amplitude of both the a- and b-waves. The results showed that the thickness of the ONL/OPL was significantly correlated with the amplitude of the a-waves in P23H rats (*R*^*2*^ = 0.884, *P* = 0.005; Fig 9A, S4 Dataset) but not with the amplitude of a-waves in SD rats (*R*^*2*^ = 0.410, *P* = 0.244; Fig 9A), whereas the thickness of the IS/OS layer was not correlated with the amplitude of the a-waves in either P23H or SD rats (*R*^*2*^ = 0.270, *P* = 0.921 and *R*^*2*^ = 0.411, *P* = 0.244, respectively; Fig 9B). In addition, the thickness of the ONL/OPL layer was significantly correlated with the amplitude of the b-waves in both P23H and SD rats (*R*^*2*^ = 0.841, *P* = 0.010 and *R*^*2*^ = 0.803, *P* = 0.040, respectively; Fig 9C, S4 Dataset), whereas the thickness of the IS/OS layer was not correlated with the amplitude of b-waves in either P23H or SD rat (*R*^*2*^ = 0.017, *P* = 0.805 and *R*^*2*^ = 0.575, *P* = 0.138, respectively; Fig 9D). A step-wise multivariate regression analysis (Tables 1 and 2) also indicated that there were significant correlations between the thickness of ONL/OPL and the amplitudes of both a-waves (*β* = −0.940, *P* = 0.005; Table 1) and b-waves (*β* = −0.917, *P* = 0.010; Table 2) in P23H rats.

**Table 1:** Multivariate regression analysis between the amplitude of a-wave (μV) and the thickness of retinal sublayers (μm) in P23H rats.

Sublayer	β	*P*
B	−0.940	0.005
A	-0.352	0.177
C	0.169	0.414
D	-0.144	0.675

Abbreviations: A, RNL + GCL + IPL + INL; B, OPL + ONL; C: IS + OS; D, RPE + choroid. β: standardized regression coefficient

**Table 2:** Multivariate regression analysis between the amplitude of b-wave (μV) and the thickness of retinal sublayers (μm) in P23H rats.

Sublayer	β	*P*
B	0.917	0.010
A	0.261	0.443
C	-0.106	0.677
D	0.075	0.855

Abbreviations: A, RNL + GCL + IPL + INL; B, OPL + ONL; C: IS + OS; D, RPE + choroid; β: standardized regression coefficient

## Discussion

In the present study, we characterized the OCT images during the natural course of retinal degeneration in rhodopsin P23H transgenic rats (line 2) and tried to identify specific relationships between OCT images and photoreceptor degeneration caused by the rhodopsin P23H mutation. Because cell death mechanisms of photoreceptor degeneration appear to be variable and depend on their causative mutations [30, 31], we need to inductively characterize the relationship between OCT images and morphologic or physiologic abnormalities during the photoreceptor degeneration induced by each causative gene mutation. Once OCT images of RP models are characterized according to the causative mutation, we will be able to apply these findings to explore the morphologic and/or physiologic changes occurring during retinal degeneration in patients with RP associated with the same mutation.

In the qualitative analysis, we found that the bands equivalent to the human EZ and IZ were recognized in the SD rats throughout the observation period [28]. However, in the P23H rats, these distinct bands only appeared until P32, and were no longer found after P46. Instead, the IS/OS layer became diffusely hyperreflective after P46. These phenomena suggest that OCT detected some abnormal optical changes that happened in the IS/OS layer. Although no apparent abnormal findings could be identified in the IS/OS layer on a histological analysis at P62, disarrangement of the disc membranes was clearly detected by an electron microscopic analysis at the same age (Figs 4 and 5). These results suggest that OCT can detect early changes occurring in the photoreceptor OS disc membranes as diffuse hyperreflective zone in the IS/OS layer, even at the stage when these abnormalities cannot be identified by histologic HE staining. In addition, the hyperreflective zone was not changed in quality throughout retinal degeneration.

Furthermore, although the histologic analyses demonstrated distinctive histological changes in the OS by comparing the findings at P62 with those at P169, there were no qualitative differences in the OCT findings of the IS/OS layer between these 2 time points. These characteristics indicate that the hyperreflective zone on OCT is a rather sensitive sign to morphological abnormalities in the OS discs but does not differentiate further progression of disarrangement and degeneration in the IS/OS layer. This may become a limitation in the qualitative analysis of OCT. Nonetheless, these morphological features in OCT may derive from early abnormal morphogenesis of the OS discs in the P23H rat, because a small part of the P23H opsin molecule is trafficked to the OS and causes abnormal disc formation [32, 33]. Furthermore, P23H rhodopsin reportedly destabilizes the OS disc membrane [34]. Abnormal disc formation was also shown in our electron microscopic analysis (Fig 5).

We previously detected a “hyperreflective band” instead of IZ in the early phase of retinal degeneration in RCS rats [25]. We speculated that this “hyperreflective band” derives from extracellular lamellar materials specifically appearing in the photoreceptor layer of RCS rats [35]. In the present study, we did not find such a “hyperreflective band”, but instead, detected the diffuse hyperreflective change in the IS/OS layer on OCT in P23H rats. We believe that the “hyperreflective band” seen in the RCS rats and the diffuse hyperreflective changes detected in the P23H rats are different entities and may be derived from the differences in the mechanisms underlying photoreceptor cell death between RCS and P23H rats. In the RCS rat, a mutation in the *mertk* gene causes defective phagocytosis in the RPE [36], resulting in consecutive photoreceptor degeneration secondary to aggregated extracellular lamellar materials, whereas the P23H mutation in the rhodopsin gene induces misfolding of the opsin molecules and aggregation of most of the misfolded proteins in the endoplasmic reticulum (ER), causing ER stress and subsequent more direct photoreceptor cell death [32, 37]. However, a small portion of the P23H rhodopsin molecules is trafficked to the outer segment, causing disarrangement of the discs [34]. Therefore, we speculate that the diffuse hyperreflective changes in the IS/OS layer that we observed in the OCT of the P23H rats may be derived from the disarrangement of the outer segment discs.

In the quantitative analysis, we found that the retinal outer layer, which mainly consisted of the ONL, gradually lost its thickness over the course of retinal degeneration. In addition, the thickness of the outer layer was significantly correlated with the amplitude of both the a- and b-waves on ERG. In contrast, the thickness of the photoreceptor IS/OS layer was relatively well maintained until P100, and gradually reduced thereafter. Furthermore, there was no statistically significant correlation between the thickness of the IS/OS layer and the amplitude of the a-wave of ERG. However, these relationships do not necessarily suggest that the deterioration of the a-wave is more dependent on the number of photoreceptor cells than on the structural deformity of the OS discs. Because the amplitude of the a-wave had already decreased by approximately 50% at P42 (Fig 7), the structural changes causing diffuse hyper-reflectance in the OS discs (Fig 2) were deemed to be related to the decreased amplitude of the a-wave. This speculation is partly supported by the previous observation reported by Sakami, et al. [33] that phototransduction mediated by P23H rhodopsin was not a major cause of retinal degeneration in the heterozygous P23H knock-in mice.

In conclusion, OCT images sensitively detected structural abnormalities in the OS discs in P23H transgenic rats, whereas photoreceptor degeneration indicated by a-wave amplitudes depended on the thickness of the ONL and OPL as well as the qualitative change in the IS/OS layer. These characteristics may be key findings that aid in analyzing photoreceptor degeneration in patients with RP associated with class II rhodopsin mutations like P23H.

## Supporting information

S1 Dataset Raw data for the retinal layer analysis. (PDF)

S2 Dataset Raw data for the amplitude of ERG a- and b-waves in P23H rats. (PDF)

S3 Dataset Raw data for the amplitude of ERG a- and b-waves in SD rats. (PDF)

S4 Dataset Raw data for the multivariate regression analysis between the amplitude of a- and b-waves and the thickness of retinal sublayers. (PDF)
