# Supplementary material for: Characterization of photoreceptor degeneration in the rhodopsin P23H transgenic rat line 2 using optical coherence tomography
Source: PLoS One. 2018 Mar 9;13(3):e0193778. doi: 10.1371/journal.pone.0193778 (PMC5844545; doi:10.1371/journal.pone.0193778)
Supplement: S1 Dataset — (PDF) [file pone.0193778.s001.pdf]

SD and P23H rats retinal layer summary 1

Sublayer<br>Layer	Inner Retinal Layer (A)<br>NFL, GGL, IPL, INL	Outer Retinal Layer (B)<br>OPL, ONL	IS/OS layer (C)<br>IS, OS	RPE + Choroid (D)<br>RPE, CHR
SD rat				
P 26				
1	87.777	84.206	32.227	35.853
2	85.092	84.481	32.162	35.724
3	90.463	83.932	32.292	35.982
mean ± SE	87.777 ± 1.550	84.206 ± 0.158	32.227 ± 0.038	35.853 ± 0.074
mean ± SD	87.777 ± 3.798	84.206 ± 0.3876	32.227 ± 0.0923	35.853 ± 0.1825
SD rat				
P 33				
1	88.784	76.345	30.374	27.332
2	96.449	81.921	22.431	22.861
3	85.366	79.373	29.228	34.829
4	80.671	70.541	32.871	31.163
mean ± SE	87.818 ± 3.323	77.045 ± 2.449	28.726 ± 2.232	29.046 ± 2.568
mean ± SD	87.818 ± 6.646	77.045 ± 4.899	28.726 ± 4.464	29.046 ± 5.135
SD rat				
P 54				
1	88.463	62.639	32.492	33.127
2	83.264	77.524	37.658	39.243
3	90.236	73.779	36.199	35.156
mean ± SE	87.321 ± 2.092	71.314 ± 4.470	35.450 ± 1.538	35.842 ± 1.799
mean ± SD	87.321 ± 3.624	71.314 ± 7.743	35.450 ± 2.663	35.842 ± 3.115
SD rat				
P 82				
1	73.090	73.400	41.552	51.048
2	77.189	71.898	39.015	49.896
3	68.991	74.893	44.089	52.201
mean ± SE	73.090 ± 2.367	73.397 ± 0.865	41.552 ± 1.465	51.048 ± 0.665
mean ± SD	73.090 ± 5.797	73.397 ± 2.1179	41.552 ± 2.537	51.049 ± 1.153
SD rat				
P 134				
1	72.677	69.898	43.600	52.398
2	68.492	70.172	44.177	42.135
3	76.861	69.623	43.022	62.661
mean ± SE	72.677 ± 2.416	69.898± 0.158	43.600 ± 0.333	52.398 ± 5.925
mean ± SD	72.677 ± 4.185	69.898 ± 0.275	43.600 ± 0.578	52.398 ± 10.263
SD rat				
P 247				
1	69.244	63.714	40.027	51.077
2	70.956	61.743	44.148	55.084
3	68.028	56.411	45.286	49.779
4	82.811	62.940	40.232	48.847
mean ± SE	72.760 ± 3.408	61.202 ± 1.648	42.423 ± 1.345	51.197 ± 1.374
mean ± SD	72.760 ± 6.8074	61.202 ± 3.295	42.423 ± 2.691	51.197 ± 2.748

Sublayer<br>Layer	Inner Retinal Layer (A)<br>NFL, GGL, IPL, INL	Outer Retinal Layer (B)<br>OPL, ONL	IS/OS layer (C)<br>IS, OS	RPE + Choroid (D)<br>RPE, CHR
P23H rat				
P 15				
1	100.425	93.681	29.394	32.823
2	102.317	86.748	26.858	33.340
3	103.795	89.622	26.716	42.589
4	100.919	93.094	31.283	34.503
mean ± SE	101.864 ± 0.758	90.786 ± 1.617	28.562 ± 1.096	35.814 ± 2.286
mean ± SD	101.864 ± 1.516	90.786 ± 3.234	28.562 ± 2.191	35.814 ± 4.571
P23H rat				
P 19				
1	84.518	77.399	32.000	26.703
2	89.764	80.228	29.368	31.401
3	87.514	78.221	32.075	29.748
mean ± SE	86.265 ± 1.519	78.616 ± 0.840	31.138 ± 0.890	29.284 ± 1.376
mean ± SD	86.265 ± 2.632	78.616 ± 1.455	31.138 ± 1.542	29.284 ± 2.383
P23H rat				
P 26				
1	78.613	71.704	26.655	30.683
2	76.804	73.593	33.693	31.726
3	65.672	90.497	32.565	28.561
mean ± SE	73.696 ± 4.046	78.598 ± 5.974	30.971 ± 2.182	30.323 ± 0.931
mean ± SD	73.696 ± 7.007	78.598 ± 10.348	30.971 ± 3.780	30.323 ± 1.612
P23H rat				
P 32				
1	73.355	67.75	24.544	32.302
2	83.313	65.673	27.268	35.202
3	83.452	60.962	33.367	28.474
4	83.940	64.842	32.213	30.101
mean ± SE	81.015 ± 2.557	64.807 ± 1.420	29.348 ± 2.077	31.413 ± 1.305
mean ± SD	81.015 ± 5.114	64.807 ± 2.640	29.348 ± 4.154	31.413 ± 3.197
P23H rat				
P 40				
1	66.815	67.136	33.877	30.076
2	74.910	66.452	35.058	34.546
3	78.484	68.874	35.722	30.938
mean ± SE	73.403 ± 3.452	67.487 ± 0.721	34.886 ± 0.540	31.853 ± 1.369
mean ± SD	73.403± 5.978	67.487 ± 1.249	34.886 ± 0.934	31.853 ± 2.371
P23H rat				
P 46				
1	84.217	68.733	30.600	37.000
2	80.152	67.289	38.580	32.064
3	77.895	67.980	40.250	30.544
mean ± SE	80.755 ± 1.850	68.001 ± 0.417	36.477 ± 2.978	33.203 ± 1.949
mean ± SD	80.755 ± 3.204	68.001 ± 0.722	36.477 ± 5.157	33.203 ± 3.375
P23H				
P71-72				
1	77.963	59.311	30.728	37.976
2	72.623	58.497	30.954	27.663
3	85.255	55.195	34.003	27.175
4	77.210	54.489	34.238	32.877
5	77.509	62.246	32.298	27.806
6	75.480	55.649	28.282	33.128
mean ± SE	77.673 ± 1.714	57.565 ± 1.219	31.751 ± 0.918	31.042 ± 1.757
mean ± SD	77.673 ± 4.198	57.565 ± 2.986	31.751 ± 2.248	31.042 ± 4.304
P23H rat				
P 89				
1	70.959	44.465	30.348	37.987
2	67.160	50.881	36.576	31.101
3	80.834	43.018	40.312	29.895
mean ± SE	72.984 ± 4.075	46.121 ± 2.416	35.745 ± 2.906	32.994 ± 2.520
mean ± SD	72.984 ± 7.058	46.121 ± 4.185	35.745 ± 5.034	32.994 ± 4.366
P23H				
P100-110				
1	75.910	50.808	32.535	36.233
2	83.002	46.056	34.896	35.967
3	71.6	44.672	35.71	31.516
4	77.39	38.332	46.028	31.932
mean ± SE	76.976 ± 2.354	44.967 ± 2.573	37.292 ± 2.988	33.912 ± 1.267
mean ± SD	76.976 ± 4.709	44.967 ± 5.145	37.292 ± 5.977	33.912 ± 2.535
P23H				
P125				
1	62.485	41.135	31.419	36.764
2	74.431	33.752	30.611	35.567
3	80.59	34.191	36.604	38.142
mean ± SE	72.502 ± 5.315	36.359 ± 2.391	32.878 ± 1.878	36.824 ± 0.744
mean ± SD	72.502 ± 9.205	36.359 ±4.142	32.878 ± 3.252	36.824 ± 1.289
P237-287				
1	68.409	27.166	26.904	44.724
2	70.911	23.732	30.812	82.338
3	78.748	31.023	31.252	50.749
4	57.369	27.409	28.897	55.173
mean ± SE	68.859 ± 4.418	27.333 ± 1.489	29.466 ± 0.995	58.246 ± 8.311
mean ± SD	68.859 ± 8.836	27.333 ± 2.979	29.466 ± 1.991	58.246 ± 16.622

30.803 ± 1.455
