# Supplementary material for: Characterization of photoreceptor degeneration in the rhodopsin P23H transgenic rat line 2 using optical coherence tomography
Source: PLoS One. 2018 Mar 9;13(3):e0193778. doi: 10.1371/journal.pone.0193778 (PMC5844545; doi:10.1371/journal.pone.0193778)
Supplement: S2 Dataset — (PDF) [file pone.0193778.s002.pdf]

Sup. 3 P23H Amplitudes of ERG a- and b-waves (raw data, μV)

	ID#	A-wave	B-wave
P 21-22	1	-25.500	128.000
	2	-85.000	249.786
	3	-85.877	230.449
	4	-80.500	256.000
	mean ± SD	-69.219 ± 29.241	216.059 ± 59.706
	mean ± SE	-69.219 ± 14.621	216.059 ± 29.853
P 42	1	-30.827	120.827
	2	-57.633	154.812
	3	-56.718	194.45
	4	-29.669	150.000
	mean ± SD	-43.712 ± 15.558	155.022 ± 30.272
	mean ± SE	-43.712 ± 7.779	155.022 ± 15.136
P 99	1	-28.549	147.048
	2	-46.308	166.308
	3	-39.315	128.315
	mean ± SD	-38.057 ± 8.946	147.224 ± 18.997
	mean ± SE	-38.057± 5.165	147.224 ± 10.968
P 158	1	-13.616	58.102
	2	-30.000	116.250
	3	-17.751	54.543
	mean ± SD	-20.456± 8.520	76.298 ± 34.645
	mean ± SE	-20.422 ± 4.919	76.298 ± 20.000
P 253	1	-18.500	91.500
	2	-26.500	97.000
	3	-24.500	111.500
	mean ± SD	-23.167 ± 4.163	100.00 ± 10.332
	mean ± SE	-23.167 ± 2.404	100.00 ± 5.965
P 294	1	-13.32	38.320
	2	-8.622	43.122
	3	-26.016	110.016
	4	-25.005	95.000
	mean ± SD	-18.241 ± 8.621	71.615 ± 36.247
	mean ± SE	-18.241 ± 4.310	71.615 ± 18.124
