# Supplementary material for: Characterization of photoreceptor degeneration in the rhodopsin P23H transgenic rat line 2 using optical coherence tomography
Source: PLoS One. 2018 Mar 9;13(3):e0193778. doi: 10.1371/journal.pone.0193778 (PMC5844545; doi:10.1371/journal.pone.0193778)
Supplement: S3 Dataset — (PDF) [file pone.0193778.s003.pdf]

SD rat ERG amplitudes of a- and b-waves

age	ID #	a-wave	b-wave
P 22	1	-107.214	338.714
	2	-88.946	265.446
	3	-73.385	240.385
	mean ± SD	-89.848 ± 16.933	281.515 ± 51.096
	mean ± SE	-89.848 ± 9.776	281.515 ± 29.500
P 65	1	-107.437	301.337
	2	-88.523	255.023
	3	-122.226	338.226
	mean ± SD	-106.062 ± 16.894	298.195 ± 41.690
	mean ± SE	-106.0618 ± 9.84026	298.195 ±24.070
P 85	1	-110.000	306.000
	2	-65.070	195.070
	3	-89.660	172.270
	4	-62.750	107.75
	mean ± SD	-81.870 ± 22.359	195.273 ± 82.563
	mean ± SE	-81.870 ± 11.180	195.272 ± 14.166
P 92	1	-62.688	241.688
	2	-53.29	175.290
	3	-72.233	202.233
	4	-66.018	191.018
	mean ± SD	-63.5573 ± 7.90561	202.557 ± 28.331
	mean ± SE	-63.5573 ± 3.9528	202.557 ± 14.166
P 112	1	-69.000	188.915
	2	-55.515	166.515
	3	-45.75	174.25
	4	-76.498	267.514
	mean ± SD	-61.691 ± 13.723	199.299 ± 46.416
	mean ± SE	-61.691 ± 6.861	199.299 ± 23.208
P 247	1	-33.311	123.311
	2	-38.311	168.311
	3	-54.865	215.575
	4	-68.892	220.892
	mean ± SD	-48.845 ± 16.2315	187.022 ± 45.723
	mean ± SE	-48.845 ±8.1157	187.022 ± 22.861
