# Supplementary material for: Characterization of photoreceptor degeneration in the rhodopsin P23H transgenic rat line 2 using optical coherence tomography
Source: PLoS One. 2018 Mar 9;13(3):e0193778. doi: 10.1371/journal.pone.0193778 (PMC5844545; doi:10.1371/journal.pone.0193778)
Supplement: S4 Dataset — (PDF) [file pone.0193778.s004.pdf]

S4. Raw Data for Correlation Analysis between the Amplitude of ERG a- and b-waves and the Thickness of Retinal Sublayers

P23H rats

PN day	numbers of rats	ERG a-wave	ERG b-wave	PN day	numbers of rats	Subleyer A	Sublayer B	Sublayer C
21-22	4	-69.219	216.059	19	3	86.265	78.616	31.138
42	4	-43.712	155.022	40	3	73.403	67.487	36.477
99	3	-38.057	147.224	100-110	4	76.976	44.967	37.292
158	3	-20.456	76.298	125	3	72.502	36.359	32.878
253	3	-23.167	100.00	237	2	69.660	25.258	28.858
294	4	-18.241	71.615	294	2	75.116	25.277	33.345

SD rats

PN day	numbers of rats	ERG a-wave	ERG b-wave	PN day	numbers of rats	Sublayer B	Sunlayer C
22	3	-89.848	281.515	26	3	84.206	32.227
65	3	-106.062	195.273	54	3	71.314	35.449
85	4	-81.870	202.557	82	3	73.397	41.557
112	4	-61.691	199.299	134	3	69.898	43.600
247	4	-48.845	187.022	247	4	61.202	42.423

Sublayer D
29.284
33.203
33.912
36.824
63.528
75.477
